# Evaluation of the prevalence and economic burden of adverse drug reactions presenting to the medical emergency department of a tertiary referral centre: a prospective study

**DOI:** 10.1186/1472-6904-7-8

**Published:** 2007-07-28

**Authors:** KJ Patel, MS Kedia, D Bajpai, SS Mehta, NA Kshirsagar, NJ Gogtay

**Affiliations:** 1 Departments of Clinical Pharmacology and Medicine Seth GS Medical College and KEM Hospital Parel, Mumbai 400012, India

## Abstract

**Background:**

Adverse drug reactions (ADRs) are now recognized as an important cause of hospital admissions, with a proportion ranging from 0.9–7.9%. They also constitute a significant economic burden. We thus aimed at determining the prevalence and the economic burden of ADRs presenting to Medical Emergency Department (ED) of a tertiary referral center in India

**Methods:**

A prospective, observational study of adult patients carried out over a 6 week period in 2005. The prevalence of ADRs, their economic burden from the hospital perspective, severity, and preventability were assessed using standard criteria.

**Results:**

A total 6899 patients presented during the study period. Of these, 2046 were admitted for various reasons. A total of 265/6899 patients had ADRs (3.84 %). A total of 141/265 was admitted due to ADsR, and thus ADRs as a cause of admissions were 6.89% of total admissions. A majority (74.71%) were found to be of moderate severity. The most common ADRs were anti-tubercular drug induced hepatotoxicity, warfarin toxicity and chloroquine induced gastritis. The median duration of hospitalization was 5 days [95% CI 5.37, 7.11], and the average hospitalization cost incurred per patient was INR 6197/- (USD 150). Of total ADRs, 59.62% (158/265) were found to be either definitely or potentially avoidable.

**Conclusion:**

The study shows that ADRs leading to hospitalization are frequent and constitute a significant economic burden. Training of patients and prescribers may lead to a reduction in hospitalization due to avoidable ADRs and thus lessen their economic burden.

## Background

Adverse Drug Reactions (ADRs) are a major problem worldwide and are one of the leading causes of mortality and morbidity in health care facilities worldwide. A landmark study by Lazarou et al found ADRs to be the fourth to sixth leading cause of death in the United States and serious ADRs accounted for 6.7% of hospitalized admissions. [1] A study by Ramesh et al in India carried out in a tertiary referral center in South India showed that admissions due to ADRs accounted for 0.7% of total admissions and deaths due to ADRs accounted for 1.8% of total ADRs. [2] The study also showed that the average cost involved in treating these ADRs was INR 690/- (USD 15$) per patient. Many such studies were either limited to individual units such as geriatric care [3,4] or were carried out retrospectively [5,6]. In India, very few studies have looked at ADRs as the cause of hospital admissions and fewer still have looked at costs associated with ADRs. The present study was envisaged to evaluate the prevalence of patients presenting with ADRs to the Emergency Department (ED) and to assess the causality, avoidability, and severity. The study also aimed determining the economic burden of ADRs from a hospital perspective.

## Methods

The study protocol was approved by the institutional ethics committee and the study was conducted from 1^st ^May to 15^th ^June 2005 at the King Edward Memorial hospital, an 1800 bedded tertiary referral center in Mumbai (Bombay), India. The study was observational, non-interventional, prospective and carried out in the medicine division of the ED. Only adults (age > 18 years) were studied. The 24 hours emergency department of the hospital is manned by senior lecturers who work in 8 hour shifts for a three month tenure. They work under the supervision of author 4. They form the first contact point for all patients. For the present study, authors 1, 2 and 3 each worked 8 hour shifts by rotation alongside and assessed the ADRs with them. Decision making as to whether or not an event was causally related to a drug was made either by the 3 senior lecturers or author 4, all of whom have a postgraduate degree in internal medicine and several years of experience working in the ED. While no structured questionnaire was used for the present study, drug intake formed an important component of history taking.

The definition of an ADR used was the one developed by the World Health Organization [7]. The assessment of causality was then performed for all the cases using the Naranjo's algorithm [8] The severity of ADRs was determined by using the Modified Hartwig and Seigel scale [9]. The avoidability of ADRs was assessed by using the definitions developed by Hallas et al [10] and ADRs were classified as definitely avoidable, possible avoidable and unavoidable. All the above scales were used at any given time by one senior lecturer only in view of their work timings. The methodology used was identical to the study carried out by Pirmohamed et al that looked at ADRs as the cause of admissions to hospitals in the United Kingdom. [11] Whenever ADRs were the cause of admissions, the total length of hospital stay was also recorded. The economic burden to the hospital was then calculated by the product of total number of admission days of all patients admitted with the ADR and hospital expenditure per day.

### Statistical Analysis

Descriptive statistics were primarily used and data expressed as percentages and medians with 95% CIs.

## Results

### Demographic Data

During the 6 week study period, 6899 patients presented to the Emergency Department. Of these, 2046 patients were admitted for various reasons. A total of 265/6899 patients had 340 ADRs giving 3.84% as the percentage of patients presenting to the ED with an ADR. A total of 141/265 were admitted due to the ADR, and thus ADRs as a cause of admissions were 141/2046 or 6.89% of total admissions. The mean age of patients with ADRs was 40 years. Using the Naranjo's algorithm, of the total 340 ADRs it was found that 13 were definite, 292 were probable, and 35 were possible. No change in causality was made after the initial assessment.

### Severity

Using the Modified Hartwig et al criteria, it was seen that 49/265 (18.49%) patients had mild ADRs while 198/265 (74.71%) patients had ADRs of moderate severity and 18/265 (6.79%) patients had severe ADRs. Thus a majority of the ADRs detected were of moderate severity.

### Avoidability

It was seen that 27 (10.19%) ADRs were definitely avoidable while 131 (49.43%) were possible avoidable and 107 (40.37%) were unavoidable. Thus, 59.62% (158/265) of the total ADRs detected were found to be either definitely or potentially avoidable.

### Economic burden to the Hospital

The median hospital stay of patients with ADRs was 5 days (95% CI 5.37 to 7.11) and the average cost per patient hospitalized with an ADR was INR 6,197/- (USD 150$). The total cost to the hospital due to hospitalization of patients presenting with ADRs over the 6 week period in the emergency department was INR 11,21,657/- (USD 27358).

### Commonest ADRs, Drug Classes and Organ Systems (table 1)

**Table 1:** Table depicting drugs and nature of adverse events seen during the study

Drug Class/Drug	**NO. (%) OF ADRS**	Individual drugs	Adverse Event
Anti tubercular agents (AKT)	52	HRZ (35), AKT (7), Rifampin (4), Ethambutol (2), Pyrazinamide (2) Streptomycin (1), Isoniazid(1),	Thrombocytopenia, Hepatitis, Hepatic encephalopathy, Retro bulbar neuritis, Cholestatic jaundice, Erythema multiforme, Vestibular toxicity, Gastritis, Abdominal/Respiratory/Flu like syndrome, Hypersensitivity reaction, Psychosis, Peripheral neuropathy, dysglycemia
Antiepileptics	36	Phenytoin(24), Valproate (5), Phenobarbiturate(4) carbamazepine (3)	Erythema multiforme, Symptomatic toxic carbamazepine/phenytoin/valproate levels with cerebellar signs
Antimalarials	30	Chloroquine (29), Sulfadoxine-pyrimethamine (1)	Gastritis, Giddiness, Erthyema multiforme, Psychosis, Hypersensitivity reactions, Hypotension
Anticoagulants	25	Warfarin (24), Heparin (1)	GI bleeding, haematuria, high INR, Intracranial bleed
Oral hypoglycemic agents (OHA)	16	Glibenclamide (10), Glyburide (6)	Hypoglycemia, seizures,
NSAIDS	14	Paracetamol (6), Aspirin (3), Ibuprofen (1), Etoricoxib (1), Nimesulide (2), Diclofenac (1)	Hypersensitivity reaction, Gastritis, mucosal damage, Hematuria, (Etoricoxib) potentiates OHA induced Hypoglycemia, ARF on CRF, Thrombocytopenia
Antihypertensives	10	Atenolol (4), ACE inhibitors (2), Losartan (2), Clonidine (1), Isosorbide dinitrate (1)	Hypotension, Hypersensitivity reaction, AV block, Bradycardia, Hyperkalemia, Missed dose of clonidine leading to rebound hypertension, dry mouth
Cytotoxic drugs	9	Methotrexate (3), 5-Fluorouracil (2), Cisplatin (1), Etoposide (1), Paclitaxel (1), Chemo (1)	Bone marrow suppression (with opportunistic infection), Nephrotoxicity, Liver damage, GI mucositis, Anorexia, Myelodysplastic syndrome, Alopecia, Hyperuricemia, Abdominal pain, Neutropenia
Steroids	9	Prednisolone (7), Betamethasone (2)	Gastritis, Hypertension, Hyperglycemia, Mania, Fragile skin, Facial puffiness, Osteoporosis, Opportunistic infections
Insulin	8	Humulin70/30 (8)	Hypoglycemia, Erythema/swelling/stinging at injection site
Digoxin	8	--	Symptomatic toxic digoxin levels
Fluoroquinolones	8	Ciprofloxacin(4), Ofloxacin (2) Gatifloxacin (2)	Complex partial seizures, Peripheral neuropathy, Dystonia, Hypersensitivity reaction, tendinitis, dysguesia
B lactam antibiotics	8	Amoxicillin(3), Ampicillin(2), Cefadroxil (1), Ceftriaxone (1), Ceftazidime (1)	Hypersensitivity reaction, Fixed drug eruptions, Diarrhea, muscle spasm, Generalized tonic-clonic seizure
Antiretrovirals	4	Efavirenz (2), Stavudine (1), Zidovudine (1)	Metabolic acidosis, Drowsiness, hypokalemia, Peripheral neuropathy
Benzodiazepines	4	Chlordiazepoxide (2), Diazepam (2)	Increased appetite and weight gain, Drowsiness, Ataxia, Amnesia, Vertigo
Bronchodilators	4	Salbutamol (3), Theophylline (1)	Tremors
Haematinics	3	Ferrous fumarate(2) Ferrous sulfate (1)	Epigastric pain, Heart burn, Vomiting, Metallic taste in mouth
Fibrinolytics	2	Urokinase (2)	Hypotension, AV block
Miscellaneous	15	Metronidazole (1) Nicotinic acid (1) Fluconazole (1) Ethamsylate (1) Amitriptyline (1) Cotrimoxazole (1) Oxybutinin HCl (1) Dextropropoxy- phene (1) Urograffin (1) Amikacin (1) Benzthiazide (1) Amiodarone (1) Levodopa (1) Alendronate (1) Clozapine (1)	Gastritis Facial flush, Hypersensitivity reaction (4) Thrombocytopenia, dry mouth Perioral tingling numbness, Carpopedal spasm, Cochlear toxicity Muscle Cramps, Paresthesias raised liver functions Peak dose dyskinesias Esophagitis, constipation, seizure

Anti-tubercular drug induced hepatitis, warfarin toxicity and chloroquine induced gastritis were the commonest ADRs seen accounting for 28, 24, and 22 ADRs respectively out of the 265 ADRs. Among the drug classes it was found that anti-tubercular agents were most commonly implicated and accounted for 52 ADRs. Of these 52, there were 9 severe, 42 moderate and 1 was a mild ADR. This was followed by antiepileptic and anti-malarial agents accounting for 36 ADRs and 30 ADRs respectively. Of ADRs caused by antiepileptic drugs, there were 3 severe, 27 moderate and 6 mild ADRs. The severity assessment of ADRs caused by anti-malarial agents showed that there were 17 mild, 13 moderate and no severe ADRs.

### Deaths due to ADRs

These were hepatotoxicity due to anti tubercular drugs (9), intracranial bleed due to warfarin (5), bone marrow suppression due to phenytoin (1), and hypoglycemia due to glibenclamide (2). The overall incidence of fatal ADRs was 0.83% (17/2046) of hospitalized patients.

## Discussion and Conclusion

The present prospective study carried out over a 6 week period in a tertiary referral center among adult patients showed that ADRs accounted for 3.84% (265/6899) of patients presenting to the ED. Of these 141 were admitted and thus 53.21 % (141/265) of total patients presenting to the ED with ADRs were hospitalized. At an average cost of USD 150 per patient hospitalized, they also constituted a significant economic burden.

Several studies carried out in different parts of the world have also yielded more or less similar results. The present study modeled on the study by Pirmohamed et al done in the United Kingdom had similar findings. [11] Their study found 6.5% of ADRs causing admissions, as against our finding of 6.89%. Dormann et al evaluated the economic impact of readmissions and ADRs in internal medicine. [12] ADRs were found to cause hospitalization in 6.2% of first admissions and in 4.2% of readmissions. Fattinger et al in their study in two Swiss departments of internal medicine found ADRs to account for 3.3% of all hospitalizations. [13] In the present study, the mean duration of hospitalization ranged from 5–7 days while Pirmohamed found the hospitalization to range from 4–18 days. The latter also found a larger number of definite and potentially avoidable ADRs compared to our study (72% versus 59.62%). The study by Dormann found 44.3% of ADRs to be preventable using the Schumock algorithm.

A comparison of deaths due to ADRs showed that while we had 0.83% of hospitalized patients dying because of ADRs, the study by Pirmohamed had 0.15% of hospitalized patients dying of ADRs. The study by Lazarou had 0.14% deaths that could be attributed to ADR related admissions. These differences could perhaps be due to the type of hospital, nature of disease and thus drugs prescribed and perhaps inter country differences in susceptibility.

The drawbacks of our study include the short duration (6 weeks), restriction to the medicine ED only; identification of ADRs by one senior physician only at any given time (who was on duty during rotation shifts in the ED), restriction of the study only to adults and calculation of costs based on duration of hospitalization only, which could give an underestimate of the costs associated with treating ADRs. Causality assessment was done at the point of presentation to the ED and was not changed subsequently. Thus 35 "possible" ADRs where other factors could account for the reaction were also included in the final analysis. Also, causality assessment was done independently by physicians on duty and their assessments may not have been similar to one another. [14]

A similar study by Sanchez Cuervo et al, albeit retrospective and carried out over a 1 year period in Spain showed some differences among the type of drugs causing ADRs in the hospital's ED [15] While we found anti-tubercular drug induced hepatotoxicity, chloroquine gastritis and warfarin toxicity to be the common ADRs, they found insulins, diuretics, digoxin and oral anti diabetics to be the common causes of ADRs. For a country like India anti tubercular toxicity could have additional ramifications like the problem of multi drug resistance and exposure to potentially more toxic second-line agents. Wu et al evaluated outpatient ADRs leading to hospitalization and found that anti-diabetics, anti-convulsants, anti-coagulants, beta blockers and ACE inhibitors to be the common causes of ADRs. [16] Both studies also found that ADRs occurred in older patients, while we found the mean age of patients with ADRs in our study to be 40 years.

Ayani I et al studied the economic burden in Spain of the minimum direct costs to the public health system of diagnosing and treating patients in an ED with a suspected ADR using the WHO definition of an ADR in a single month. The cost amounted to 42,732 Ecus and considering that 40% of ADRs were avoidable, they proposed that if pharmacovigilance activities included cost analysis, significant savings would result. [17] The study by Wu et al calculated the mean cost of treating an ADR per patient to be USD 9491 with 50% of this cost being the hospitalization or room charges alone. We included in our study the hospitalization or room/bed charges only as we decided to restrict the economic perspective to the hospital only. Our hospital being a public hospital caters to the lower socio-economic strata and hence costs would be different from that of a private hospital. If we assume that similar to the study by Wu et al, these represent only 50% of the total costs, the economic burden of ADRs would be considerable. Also, we found approximately 59.62% of ADRs in our study to be avoidable or potentially avoidable. This is similar to the findings of a meta-analysis where the rate of preventable ADRs was found to be 59% (inter quartile range 50–73%). [18] If these are minimized, it would lead to considerable savings.

In conclusion, ADRs are an important cause of visits to the hospital emergency department as well as an important cause of admission and thus a significant economic burden. It is likely that many of them particularly the avoidable and potentially avoidable ones may be minimized by patient and physician education and better prescribing practices and thus lead to considerable cost savings.

## Competing interests

The author(s) declare that they have no competing interests.

## Authors' contributions

KJP, MSK, and DB implemented the study in 8 hours shifts. SSM and NJG conceived of the study, designed it, co-ordinated and obtained ethics committee approval. SSM and NJG also analyzed the study, performed the statistical analysis and wrote the manuscript. NAK participated in the final analysis, interpretation and preparation of the manuscript. All authors read and approved the final manuscript.

## Pre-publication history

The pre-publication history for this paper can be accessed here:


